# Metabolic and Transcriptional Reprogramming in Developing Soybean (*Glycine max*) Embryos

**DOI:** 10.3390/metabo3020347

**Published:** 2013-05-14

**Authors:** Eva Collakova, Delasa Aghamirzaie, Yihui Fang, Curtis Klumas, Farzaneh Tabataba, Akshay Kakumanu, Elijah Myers, Lenwood S. Heath, Ruth Grene

**Affiliations:** 1 Department of Plant Pathology, Physiology, and Weed Science, Virginia Tech, Blacksburg, VA, USA; E-Mails: rainfyh@vt.edu (Y.F.); grene@vt.edu (R.G.); 2 Genetics, Bioinformatics and Computational Biology Program, Virginia Tech, Blacksburg, VA, USA; E-Mails: delasa@vt.edu (D.A.); curtisk@vt.edu (C.K.); ESM2310@vt.edu (E.M.); 3 Department of Computer Science, Virginia Tech, Blacksburg, VA, USA; E-Mails: fstaba2@vt.edu (F.T.); heath@vt.edu (L.S.H.); 4 Huck Institutes of the Life Sciences, Penn State University, University Park, PA, USA; E-Mail: auk262@psu.edu

**Keywords:** central carbon and nitrogen metabolism, plant metabolic engineering, RNA sequencing, seed storage compounds, soybean, systems biology, transcriptomics, untargeted and targeted metabolomics

## Abstract

Soybean (*Glycine max*) seeds are an important source of seed storage compounds, including protein, oil, and sugar used for food, feed, chemical, and biofuel production. We assessed detailed temporal transcriptional and metabolic changes in developing soybean embryos to gain a systems biology view of developmental and metabolic changes and to identify potential targets for metabolic engineering. Two major developmental and metabolic transitions were captured enabling identification of potential metabolic engineering targets specific to seed filling and to desiccation. The first transition involved a switch between different types of metabolism in dividing and elongating cells. The second transition involved the onset of maturation and desiccation tolerance during seed filling and a switch from photoheterotrophic to heterotrophic metabolism. Clustering analyses of metabolite and transcript data revealed clusters of functionally related metabolites and transcripts active in these different developmental and metabolic programs. The gene clusters provide a resource to generate predictions about the associations and interactions of unknown regulators with their targets based on “guilt-by-association” relationships. The inferred regulators also represent potential targets for future metabolic engineering of relevant pathways and steps in central carbon and nitrogen metabolism in soybean embryos and drought and desiccation tolerance in plants.

## 1. Introduction

Seeds are an important source of food, feed, biodiesel, and chemicals, because they are rich in oils, proteins, and carbohydrates [1,2]. These products are referred to as seed storage reserves, as they represent carbon-, nitrogen-, and energy-rich molecules needed for seed germination before the onset of photosynthesis in seedlings [3,4,5]. Oil and carbohydrates provide carbon and energy, while the seed storage proteins are the major source of nitrogen during germination. Typically, soybean seeds contain about 18% of oil (triacylglycerols), 42% of protein (2S albumins and 7S and 11S globulins), and other components, including carbohydrates, and storage and cell-wall polysaccharides, comprising the rest of the seed biomass [1,6] . However, seed composition is a variable trait. For instance, oil content can vary from 6.5% to 28.7% in dry soybean seeds depending on the plant genetic background and growth conditions [2], suggesting that central carbon metabolism leading to the accumulation of seed storage compounds in developing seeds is quite flexible and should be amenable to metabolic engineering.

Seed development in general encompasses a chronological series of developmental, metabolic, and physiological processes, controlled by relevant, and only partially understood, regulatory events. In soybean, these processes take place during the reproductive R3 (beginning pod) through R8 (full maturity) developmental stages at the whole plant, organ, tissue, cell, and molecular levels [6,7,8]. Development and metabolism in an oilseed such as soybean is relatively “simple” compared to events taking place in other parts of the plant, since the sole purpose of the seed is species propagation. This translates into the involvement of three main processes during seed development that maintain seed viability until germination. These processes include: (i) the development of proper cell and tissue identity during cell differentiation and maintaining genetic information about the species, (ii) the accumulation of reserves to fuel seed germination, and (iii) the ability to withstand unfavorable conditions that the seed encounters prior to germinating.

The early stages of embryo development (R3 reproductive stage) involve predominantly cell division and differentiation, starting with cell division in the fertilized egg, followed by a combination of highly regulated cell division and differentiation processes during tissue and organ formation [6,8]. These processes require a constant active supply of maternally provided oxidizable substrates before the embryonic cells become fully differentiated and photosynthetically active [9,10]. Nutrient supply by maternal source tissues continues even in green seeds, as they show a very active photoheterotrophic type of metabolism [11,12,13,14,15]. During this seed-filling stage (R4–7 reproductive stages), cell elongation and accumulation of seed storage compounds represent the major developmental and metabolic processes [11,16,17,18,19]. As the water content decreases and the seed biomass increases during late stages of seed filling, hormonally-regulated seed desiccation- and dormancy-related processes become active [20,21,22,23].

Each of these processes and their transitions are highly regulated and coordinated at multiple levels. This complicates the development of metabolic engineering strategies for altering the levels of seed storage compounds and their composition to our advantage and for engineering drought-resistant plants by exploring molecular basis for seed desiccation tolerance. The three stages of embryo development all involve central carbon and nitrogen metabolism. As such, the potential candidate seed-filling-specific metabolism and seed desiccation and dormancy genes targeted for metabolic engineering of seed storage compounds and drought tolerance need to be distinguished from those involved in early embryogenesis. We were interested in obtaining a systems biology perspective of transcriptional and metabolic reprogramming in developing soybean embryos to capture these transitions with associated genes. Here we present and discuss results from a detailed untargeted and targeted metabolomic and comprehensive transcriptomic time-course experiment that encompasses these transitions in developing soybean embryos. We used extensive computational analyses to integrate these diverse temporal datasets to identify unique metabolite and gene expression patterns. This allowed gaining a better understanding of transitions between these developmental stages and identifying seed filling- and desiccation tolerance-specific genes.

## 2. Results and Discussion

### 2.1. Metabolic Reprogramming in Developing Soybean Embryos

#### 2.1.1. Lipid and Protein Accumulation in Developing Soybean Embryos

**Figure 1:**
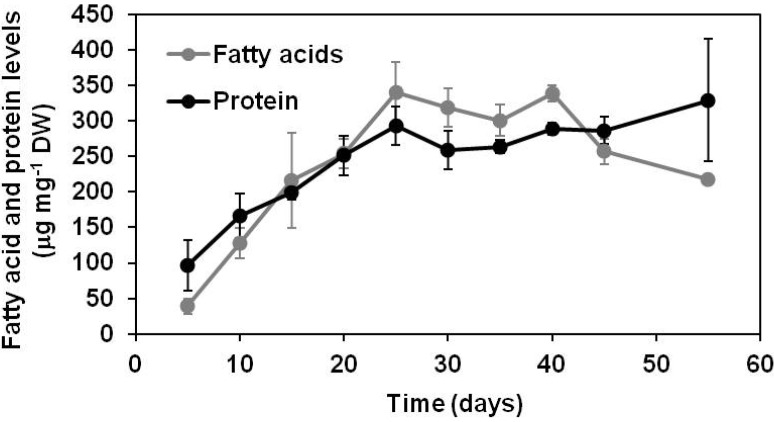
Changes in fatty acid and protein levels in developing soybean embryos. Fatty acids from hydrolyzed lipids and proteins were analyzed by GC-FID as fatty acid methylesters and by a fluorescent hydrophobic protein assay, respectively, as described in the Experimental Section. Young 5-day-old embryos already accumulate seed storage compounds and the levels of these compounds gradually increased in developing soybean embryos from day 5 to 25.

Soybean plants at reproductive stages R3 and 4 started to produce pods 7 to 10 days after anthesis. During the reproductive growth stage R5 (additional 5–7 days after anthesis), soybean plants carried pods containing young green seeds (3 mm, day 0 of the time course) that had already started to accumulate seed storage oils and proteins. We followed changes in the levels of proteins and lipid-derived fatty acids during seed filling, starting with young embryos (time point 5 days, corresponding to 17- to 22-day-old embryos) and ending with maturing and desiccating embryos (time point 55 days, corresponding to 67- to 72-day-old embryos). The accumulation of these major seed storage compounds increased in a nearly linear manner in developing soybean embryos until day 25 when a plateau was reached for both fatty acids and proteins. With respect to protein levels, the plateau was maintained until the end of the time course, while the levels of individual fatty acids started to decrease after day 40 in the time course in desiccating embryos (Figure 1, Table 1). Oil degradation during oilseed embryo development is well documented [24,25] and will be discussed in the context of the peroxisomal glyoxylate cycle.

Fatty acids analyzed in this study can originate from membrane lipids or storage oil (triacylglycerol). During early stages of soybean embryo development, the oil accumulation just started and the majority of fatty acids most likely originated from membrane lipids. At the later stages of development and in desiccating embryos, fatty acids are derived predominantly from oil. Williams 82 used in this study and Harosoy 63 used in [26] accumulate normal oil levels and similar trends in oil accumulation can be expected. At 30 days after anthesis, Harosoy 63 accumulated 14% of oil [26], while Williams 82 embryos about 20% (time point at day 15 corresponds to 27–32 days after anthesis). At 40 days after anthesis, Harosoy 63 accumulated 21% of oil [26], while Williams 82 about 32% (day 25 corresponds to 37–42 days after anthesis). Forty days after anthesis represented the beginning of the plateau in both cultivars. In Harosoy 63, oil levels began to decrease in 70-day-old embryos [26], while in Williams 82 in 57- to 62-day-old embryos (day 45). Assuming similar timing of the onset of seed filling and levels of oil accumulation in these two different soybean cultivars, it appears that significant portion of fatty acids (Figure 1) originated from membrane lipids during early stages of seed filling.

Similarly, seed storage proteins dominate the biomass composition during later stages of embryo development, while young embryos contain high levels of enzymes and other proteins. However, seed storage proteins are highly hydrophobic and were targeted in this study by using a hydrophobic protein fluorescent assay (Experimental Section), so the water-soluble proteins are underrepresented. Considering other biomass components, cells of young embryos on the onset of seed filling could resemble a typical plant cell containing predominantly cell-wall components, soluble sugars, starch, soluble and membrane-bound proteins, membrane lipids, energy cofactors, nucleic acids, metabolites, and minerals [27]. However, comprehensive quantification of absolute levels of different biomass components is very challenging and our focus was on the accumulation of seed storage compounds.

#### 2.1.2. Polar Metabolomics in Developing Soybean Embryos

Embryos at early stages of development are metabolically very active and accumulate a variety of polar metabolites of central carbon and nitrogen metabolism, including sugars, sugar alcohols, sugar acids, amino acids, organic amines and alcohols, carboxylic acids, and phenolic compounds. We were able to detect 55 of these various metabolites by using untargeted metabolomics via gas chromatography-mass spectrometry (GC-MS) and targeted AccQ•Tag^TM^ amino acid analysis via Waters Ultra Performance Liquid Chromatography (UPLC) coupled to fluorescent detection (FLD) (Supplementary Table 1). We used these two different, partially complementary approaches because some amino acids were not easily detected at low levels with GC-MS because they do not derivatize and analyze well as trimethylsilyl derivatives. In fact, majority of metabolites were present at high levels in young embryos and kept gradually disappearing in aging embryos. Many metabolites, particularly amino acids, could no longer be detected in maturing and desiccating embryos by GC-MS. UPLC-FLD provided a more sensitive solution than GC-MS and many amino acids could be detected even at day 55 of the time course. Though sensitive, UPLC-FLD lacks spectral specificity provided by MS, since the spectra of amino acids and organic amines derivatized with the fluorescent AccQ reagent will be virtually identical. As such, separation is crucial and the confirmation of the presence of individual amino acids in these samples by different methods is typically needed. The low levels at which these amino acids were detected in older embryos point to the satisfactory separation of these compounds by UPLC-FLD, but we cannot rule out a possibility that some low-abundance amino acids coeluted with other unidentified organic amines that may be present in our samples.

**Table 1:** Fatty acid levels in developing soybean embryos. Fatty acids from hydrolyzed lipids were analyzed by GC-FID as fatty acid methyl esters as described in the Experimental Section. Averages ± SD (μg mg^−1^ dry weight) of three biological replicates are shown for each day in the time course.

Fatty Acids									
Day	16:0	18:0	18:1 ^Δ^^9^	18:1 ^Δ^^12^	18:2 ^Δ^^9,12^	18:3 ^Δ^^9,12,15^	20:0	20:1 ^Δ^^11^	22:0
**5**	8.0 ± 2.2	2.2 ± 0.4	6.1 ± 2.3	0.45 ± 0.19	14 ± 4	9.4 ± 2.5	0.40 ± 0.06	0.12 ± 0.04	0.34 ± 0.10
**10**	18 ± 2	6.1 ± 1.0	29 ± 9	1.9 ± 0.3	57 ± 8	16 ± 1	0.80 ± 0.14	0.31 ± 0.05	0.47 ± 0.07
**15**	29 ± 9	10 ± 3	52 ± 28	3.5 ± 0.9	98 ± 24	24 ± 2	1.1 ± 0.4	0.47 ± 0.23	0.77 ± 0.26
**20**	29 ± 2	11 ± 1	69 ± 7	3.8 ± 0.3	120 ± 9	20 ± 1	1.2 ± 0.1	0.56 ± 0.06	0.77 ± 0.08
**25**	38 ± 5	16 ± 2	73 ± 10	4.2 ± 0.6	183 ± 23	25 ± 4	1.5 ± 0.2	0.72 ± 0.09	1.2 ± 0.1
**30**	35 ± 2	14 ± 1	81 ± 11	4.2 ± 0.6	161 ± 16	22 ± 1	1.4 ± 0.0	0.76 ± 0.07	1.1 ± 0.1
**35**	33 ± 2	14 ± 0	66 ± 3	3.7 ± 0.4	162 ± 20	22 ± 2	1.3 ± 0.1	0.71 ± 0.05	1.0 ± 0.0
**40**	37 ± 0	15 ± 1	81 ± 10	4.2 ± 0.4	176 ± 10	22 ± 2	1.5 ± 0.1	0.83 ± 0.10	1.2 ± 0.0
**45**	29 ± 2	11 ± 0	65 ± 4	3.2 ± 0.4	132 ± 13	16 ± 1	1.2 ± 0.0	0.67 ± 0.06	0.93 ± 0.01
**55**	25 ± 1	9.0 ± 0.7	54 ± 6	2.8 ± 0.2	112 ± 10	14 ± 1	0.95 ± 0.10	0.52 ± 0.01	0.78 ± 0.07

**Figure 2:**
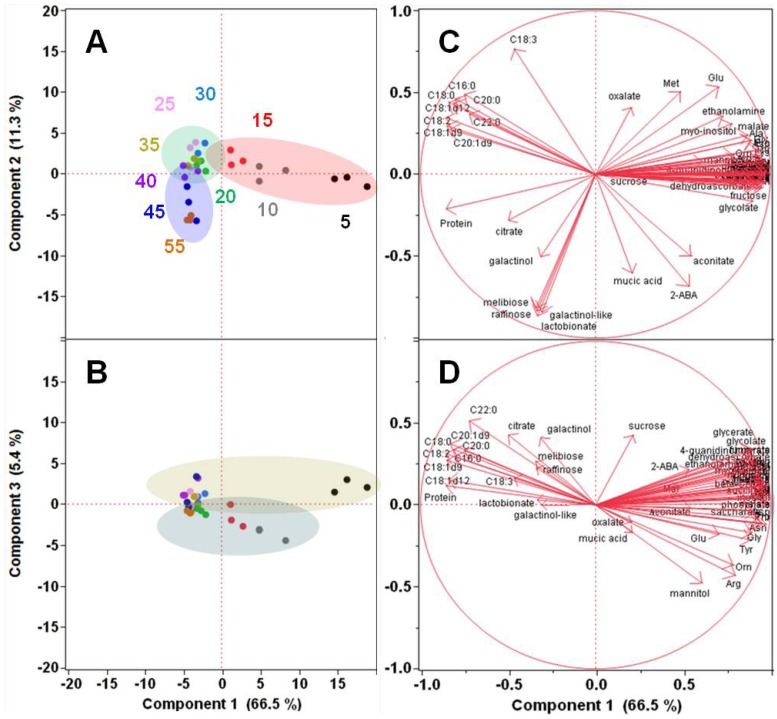
Principal Component Analysis on Metabolite Level Correlations. Score plots (A and B) and loading plots (C and D) are shown for combinations of PC1, 2, and 3. PCA was performed on combined non-redundant data involving three replicates of the relative or absolute levels of free metabolites, individual fatty acids from hydrolyzed lipids, and hydrophobic protein in developing soybean embryos by using JMP Pro 10 software (SAS, Cary, NC, USA). The color-coded numbers in A represent the corresponding age of the embryos (days 5 through 55), and each dot of the same color represents replicate samples. The color-coding is retained for B. The ovals highlight the basic clustering/separation of similar/dissimilar samples. In the loading plots C and D, the eigenvectors represented by red arrows show how (the direction) and how much (the length) each metabolite contributes to the individual correlations represented by PC1, 2, and 3.

While simple sugars and amino acids were disappearing in maturing embryos, storage and desiccation oligosaccharides (e.g., galactinol and other sugar alcohols and raffinose) showed the opposite trend and accumulated in maturing embryos (Supplementary Table 1). To obtain a global perspective on these metabolic changes, we performed principal component analysis (PCA) on correlations of metabolite levels, including fatty acids from lipids and hydrophobic proteins, in these developing soybean embryos. The first three principal components (PCs) accounted for 83.2% of the total variance among the samples (Figure 2). The early, intermediate, and late developmental stages representing different major metabolic processes could be clearly distinguished from each other based on combinations of PC1 and 2 (Figure 2A,B). Embryos at days 5, 10, and 15 were metabolically distinct from one another as well as from the rest of the embryos based on PC1. This strongest correlation was driven by the decrease in the levels of the intermediates of the central carbon and nitrogen metabolism (amino acids and monosaccharides) and the accumulation of seed storage compounds (oil and protein) in developing soybean embryos during seed filling (Figure 2C).

Embryos at days 20, 25, 30, and 35 clustered together based on both PC1 and 2, while they could be separated from those of days 45 and 55 based on PC2 (Figure 2A), suggesting that these oldest embryos (day 45 and 55) are metabolically different from embryos at days 15–40. PC2 correlation involved oligosaccharides (raffinose and melibiose) and their sugar alcohols and acids (galactinol, lactobionic acid, and an unknown galactinol-like sugar alcohol), all negatively correlating with oxalate and partially with Glu and Met (Figure 2C). Based on these observations, PC2 explains best the metabolic changes that occur during the acquisition of desiccation tolerance. Some developmental stage separation could also be observed based on PC3 and the embryos at days 10 and perhaps 15 appeared metabolically more similar to each other than to the rest of the embryos based on PC3 (Figure 2B). In PC3, eigenvectors point to a partial correlation between oligosaccharides and citrate, all negatively, strongly correlated with mannitol, Arg, and Orn and weakly with oxalate and mucic acid (Figure 2D), which is not easily interpreted. No clear data-point separation was observed based on PC4 and the rest of the PCs (not shown). Nevertheless, the first two PCs reflect very clearly the metabolic reprogramming occurring during soybean embryo development. 

**Figure 3:**
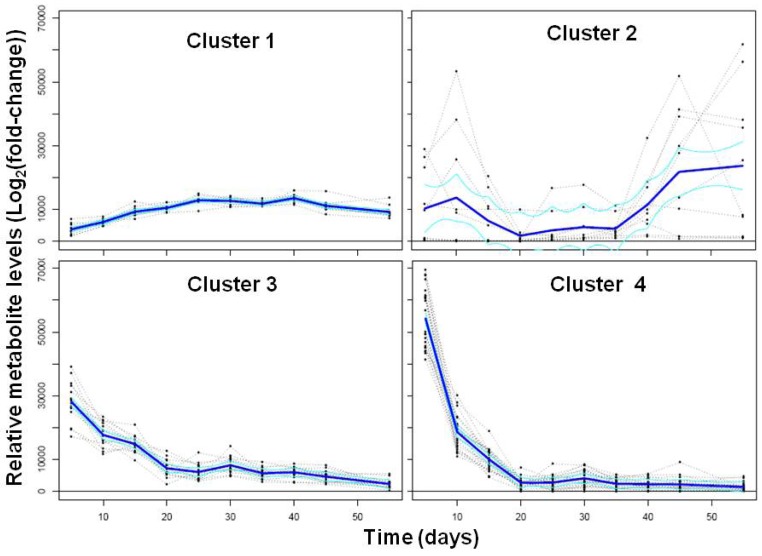
Changes in metabolite levels in developing soybean embryos. Relative metabolite levels obtained from GC-MS-FID and UPLC analyses and protein levels were subjected to Limma computational analysis to obtain trends as described in the Experimental Section. Only metabolites that showed statistically significant changes (*p*-value < 0.05) for at least one time point were subjected to SplineCluster analysis resulting in four clusters (shown in the same scale). The levels of homoserine, aconitate, mucic acid, ornithine, and Met did not change significantly over embryo development. Cluster 1 contained 10 metabolites (citrate and nine lipid-derived fatty acids that were detected by GC-FID (Table 1)) and total protein. Cluster 2 contained 10 metabolites (sucrose, oxalate, mannitol, lactobionate, melibiose, galactinol, galactinol-like, raffinose, Arg, 2-aminobutyrate). Cluster 3 contained 16 metabolites (phosphate, malate, pinitol, inositol, ethanolamine, myo-inositol, maltose, Gly, Asp, Glu, Thr, Ala, γ-aminobutyrate, Pro, Lys, Tyr). Cluster 4 contained 22 metabolites (fructose, glucose, glycolate, malonate, succinate, glycerate, fumarate, beta-cyanoAla, pyroGlu, 4-guanidinobutyrate, putrescine, dehydroascorbate, saccharate, His, Asn, Ser, Gln, citrulline, Val, Ileu, Leu, Phe).

We also used the SplineCluster tool enabling Bayesian coclustering analyses for time-series [28] to obtain a visual representation of the global trends in the profiles of 55 identified metabolites and seed storage compounds (total protein and nine lipid-derived fatty acids) during embryo development. This analysis yielded four clusters representing four major temporal trends (Figure 3). Cluster 1 mimicked the accumulation of seed storage compounds shown in Figure 1, as they all were present in this cluster in addition to citrate. Metabolites present in cluster 2 showed much more variable changes in their levels than did citrate or the seed storage compounds. Specifically, there was an overall increase from day 5 to 10, followed by a decrease from day 10 to 20, a moderate or no increase from day 20 to 35, and an increase from day 35 to 55. The majority of the metabolites present in cluster 2 were oligosaccharides and sugar alcohols, which are known to accumulate in developing seeds during desiccation [20,21,22,23,24]. Clusters 3 and 4 contained the monosaccharides, amino acids, and carboxylic acids of central carbon and nitrogen metabolism. The overall trend was similar in these two clusters, with a decrease between day 5 and 20, followed by a region of no or little change in metabolite levels at day 20 to 55. The only difference between these two clusters was the slope of the initial decreases in metabolite levels (Figure 3). In agreement with the PCA results, clustering analysis also revealed three major metabolic processes: (i) accumulation of seed storage compounds, (ii) global decrease in the levels of the intermediates of central carbon and nitrogen metabolism, and (iii) the accumulation of desiccation-related oligosaccharides and their alcohols.

### 2.2. Transcriptional Reprogramming in Developing Soybean Embryos

Different stages of oilseed embryo development are characteristic by different metabolic processes predominating specific stages. Metabolic changes in developing embryos are accompanied by the corresponding gene expression changes. To obtain a global perspective on these changes, a detailed transcriptomic time-course was performed. First, we visualized changes in steady-state transcript levels by using MapMan that was improved from the original version by enabling visualizing time-courses from within the individual bins [29,30]. Second, we performed a time-course co-expression analysis by using SplineCluster [28] as was done for metabolites. 

#### 2.2.1. RNA Sequencing-Based Transcriptomics

Based on the existing gene expression data and current bioinformatic predictions, *Glycine max* genome contains 54175 protein-encoding loci and 73320 transcripts [31]. RNA sequencing-based transcriptomics provides a much deeper level of information than microarray-based transcriptomics [32,33,34] and it was not surprising to see that the majority of the genes present in the soybean genome were expressed at least in one time point (41619 genes). Genes encoding enzymes in primary and secondary metabolism represent a small proportion of the total genes in the genome based on the current plant genome-scale flux models [27,35,36,37]. The majority of expressed genes encoded proteins of unknown function or involved in cellular processes other than seed desiccation and metabolism related to seed filling, such as embryonic program and general cell maintenance, cell growth, molecular trafficking, signaling, and regulation.

Most reads were found to map to multiple genes of highly conserved sequences. This observation agrees with the inferred paleopolyploidy of soybean, as its genome was duplicated twice, approximately 59 and 13 million years ago, resulting in the existence of multiple copies (2–6) for nearly 75% of the genes in the genome [38,39,40]. There was probably not enough time for extensive mutagenesis that could potentially lead to functional diversification of these duplicated genes and most of them are likely to have the same or similar function [41]. From the metabolic engineering perspective, this represents at least two obstacles: (i) genetic redundancy and (ii) unresolved gene function.

Based on the Cuffdiff 2 analysis, 10794 genes showed statistically significant differential expression (*p*-value cutoff of 0.05) at least one time point in developing soybean embryos. This leaves about 30000 genes that showed no statistically significant changes in their steady-state transcript levels during embryo development. Results from this analysis can be found in Supplementary Table 2. We were particularly interested in exploring the genes that showed changes in expression and the trends of the actual changes, as some of these genes could represent targets for metabolic engineering.

#### 2.2.2. Analysis and Visualization of Global Transcriptional Changes during Embryo Development

MapMan [29,30,42] was used to visualize log_2_ of fold-changes in the expression of genes relevant to primary and secondary metabolism in developing soybean embryos. From the global point of view, the major changes in gene expression in terms of the number of genes and the magnitude of the change were observed in later, as opposed to earlier stages of development (Supplementary Table 3 and Video). At day 10, the majority of metabolic genes were up-regulated relative to those at day 5, while maturing and mature embryos showed down-regulation of many central carbon and nitrogen metabolism genes that were actively being expressed in young embryos at day 5. At the same time, there was an up-regulation of many metabolism-related genes in these older embryos. This did not hold true for all processes, as there were several processes where gene expression was predominantly either up or down regulated during specific stages of embryo development.

Light reaction- and Calvin cycle-associated genes showed a progressive down regulation in aging embryos. An exception was one of the small ribulose-1,5-bisphosphate carboxylase (RUBISCO) subunits (Glyma13g00190) and Rubisco activase (Glyma03g12070), which showed gradual and consistent 4- to 8-fold and 4- to 6-fold increases, respectively, in the corresponding transcript levels during seed filling and desiccation stages. RUBISCO is known to function in photoheterotrophic central carbon metabolism in developing oilseed embryos to improve carbon-use efficiency [11,16,43,44]. Up regulation of a gene encoding RUBISCO and its activase is consistent with an active plastidic RUBISCO bypass in developing soybean embryos.

The expression of genes involved in gluconeogenesis and the peroxisomal glyoxylate cycle was up-regulated in maturing and desiccating embryos, in some instances as early as at day 35. These genes encoded malate synthase (Glyma05g03090, Glyma17g13730), NAD-dependent malate dehydrogenase (Glyma01g40580, Glyma08g06820, Glyma11g04720), phosphoenolpyruvate carboxykinase (Glyma01g02330, Glyma08g36820), citrate synthase (Glyma14g03000, Glyma02g45790), and isocitrate lyase (Glyma06g45950, Glyma12g10780). These enzymes were demonstrated to be active along with those involved in β-oxidation at later, desiccation-related stages of developing rape embryos [25]. Detailed labeling studies revealed that gluconeogenesis was not occurring in rape embryos, instead, CO_2_, malate, citrate, Asp, and Glu were formed. The peroxisomal glyoxylate pathway was proposed to provide oxidizable carboxylic acids to the mitochondrial TCA cycle to provide energy and substrates for protein synthesis, which occurs also during late desiccation stages [25]. This is in an agreement with decreased levels of fatty acids in the last two time points, which could be indicative of lipid degradation to provide carbon and energy in maturing embryos with low levels of photosynthesis and maternal sucrose supply. Rape seeds accumulate up to 60% of oil and the oil losses due to β-oxidation during desiccation stages were at least 10% [25]. Soybean seeds accumulate predominantly protein and based on our data, the losses of lipids to facilitate protein synthesis could exceed 50% of the total production. Suppressing genes involved in the glyoxylate cycle represents a potential metabolic engineering strategy for improving oil content and carbon-use efficiency in soybean seeds, but most likely at the expense of protein.

Raffinose and galactinol-related genes were also up-regulated during seed filling and desiccation (day 20–55), including several genes that were nearly identical or highly similar to Arabidopsis genes encoding galactinol-raffinose galactosyltransferase (Glyma19g40550), galactinol-sucrose galactosyltransferase (Glyma05g02510, Glyma06g18890), galactinol synthases 1 and 2 (Glyma19g40680, Glyma20g22700, Glyma03g38080, Glyma10g28610, Glyma19g41550, and Glyma03g38910), and seed imbibition O-glycosyl hydrolases (Glyma04g36410, Glyma03g29440). Up-regulation of these genes, which are involved in the synthesis of raffinose-family oligosaccharides is consistent with the accumulation of raffinose, galactinol, and related compounds observed during seed filling and desiccation phases. These compounds serve as osmoprotectants and antioxidants in leaves and developing seeds [45,46,47,48].

**Figure 4:**
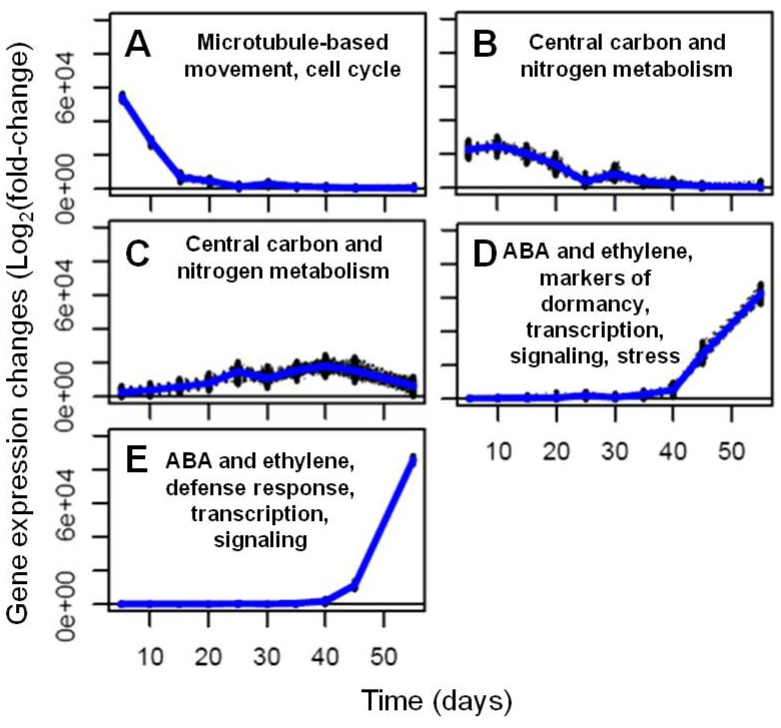
Major five gene expression trends (**A**–**E**) representing processes of interest observed in developing soybean embryos.

We also aimed to capture the transitions involving the onset and the active synthesis of seed storage compounds as well as the onset of desiccation and dormancy in developing soybean embryos. We hypothesized that capturing these transitions will enable the identification of specific genes encoding proteins involved in these different processes associated with embryo development and metabolism. Co-expression analysis using the log_2_ of fold-changes in expression of genes that showed statistically significant differences for at least one time point (10794 genes out of 41619 total expressed genes) generated 105 clusters (Supplementary Figure 1). Genes belonging to the individual clusters can be found in Supplementary Table 4. Clusters enriched in genes involved in processes of interest could be categorized into five basic gene expression patterns A–E shown in Figure 4. Clusters showing similar patterns tended to contain functionally related genes. For instance, careful analysis of clusters 10–20 (represented by trend A) revealed a strong enrichment in genes involved in microtubule-based movement and other cell cycle-associated structural and regulatory processes, including DNA replication, chromosome remodeling, mitotic cell division, cell wall remodeling, fatty acid and glycerol biosynthesis, and carbohydrate metabolism.

Genes involved in central carbon and nitrogen metabolism and metabolite transport were present in nearly all clusters, but many showed high transcript levels in the beginning of the time course, with a gradual decrease and no or low expression levels after day 25. Clusters 21–26 are representative of these expression patterns (trend B in Figure 4). These clusters were particularly enriched in genes related to metabolism and metabolite transport, including photosynthesis, respiration, carbohydrate and starch metabolism, cell wall remodeling, glycolysis, citric acid cycle, pentose phosphate pathway, lipid biosynthesis, and amino acid metabolism (Supplementary Table 4). These two groups of clusters contained genes showing similar trends - a gradual decrease in the steady-state transcript levels. On the other hand, in clusters 59–62, a corresponding sub-set of heterotrophic metabolism-related genes (photosynthesis-related genes were absent from these clusters) showed somewhat an opposite trend, as they became expressed after day 15 and showed slowly increasing transcript levels until day 50 (trend C in Figure 4, Supplementary Table 4).

Genes expressed in clusters 91–94 and 100–104 (trends D and E, respectively, in Figure 4) reflected the completion of phases in which metabolism was most active, and the onset of processes associated with stages in the induction of dormancy and the acquisition of desiccation. The information available about the soybean genes that appear in these clusters comes almost exclusively from Arabidopsis databases and hence we have analyzed the contents of clusters 89–104 using Gene Ontology (GO) categorization. In Supplemental Table 5, all clusters discussed in this section are presented including the IDs of the Arabidopsis homologs together with their respective GO categories. Many of the genes expressed during this last phase of seed development are associated with transcription and other regulatory processes. The four clusters represented by trend D show a common pattern with modest and constant expression before time point 40 and substantial increases towards the end of the time course, into the seed maturation phase. In contrast, those represented by trend E show essentially no expression until the last two time points in development, the period in which seed maturation is nearing completion, and dormancy has been fully induced. It is possible, therefore, through study of these clusters, to identify processes that are specific to these maturation phases in seed development. 

Specific processes associated with lipid metabolism appeared to have been active in the earlier phase of the seed maturation period. However, as witnessed by the up regulation of abscisic acid- (ABA) and ethylene-related processes (including ethylene and ABA signaling), dormancy induction had already begun at that time. Jasmonic acid (JA)-related signaling genes (33 genes) were also responsive during this period. Marker genes for “seed development ending in dormancy” were also observed in both groups, such as Glyma01g30670, Glyma13g39090, and Glyma11g05540, representing the homologs of AT5G01300, AT2G27300 and AT4G37300, present either in clusters 91–94 or clusters 100–104. Stress response genes associated with salt, osmotic and responses to pathogens appeared in clusters 91–94 (73 genes) and clusters 100–104 (46 genes).

A group of genes (82 in all) that fell into GO categories associated with “chloroplast” appeared in the 91–94 and 100–104 groups. Chloroplast annotations are represented even more highly considering the cellular component (CC) GO annotations, showing 51 CC in clusters 91–94, 25 CC annotations in clusters 100–104, and 75 CC annotations overall. Genes associated with the chloroplast category included genes encoding proteins involved primarily in amino acid metabolism and tocopherol biosynthesis. Tocopherols are potent antioxidants protecting membrane lipids and accumulate at high levels in oilseeds [49,50]. Tocopherols also play an important protective role during seed desiccation and longevity [51,52]. A homolog of Glyma13g03390 encoding NPQ4 (AT1G44575 in Arabidopsis), the 22kDA protein associated with non-photochemical quenching from PSII, was also a member of the 100–104 group, in which genes were only up-regulated at the last two time points. Glyma08g23540 and Glyma07g02480, two homologs of a gene associated with unfolded protein binding, AT1G80920, also formed part of the 100–104 group. A large set of genes encoding transcription factors were part of both groups (30 in the 91–94 group and 27 in the 100–104 group), especially those associated with ethylene action (9 genes in 91–94 and 11 genes in 100–104 cluster group). Transcription-associated annotations are represented even more highly when examining molecular function (MF) annotations in addition to the biological process annotations with 30 MF annotations in clusters 91–94, 27 MF annotations in clusters 100–104, and 57 overall MF annotations.

The relevant responses underlying desiccation acquisition or drought tolerance were in the past regarded as being purely stress related. In fact, in the case of developing seeds, this genetically programmed developmental process proves to be more complex than a simple acquisition of desiccation tolerance. There is good evidence from previous high-throughput studies that desiccation acquisition and dormancy induction in orthodox seeds involve a specific and active transcriptional and metabolomic program [20,22], with 30% of genes expressed during Arabidopsis seed development being expressed during this late and final stage of seed development. That same pattern is reflected in the data reported here, where 22% of all genes expressed over the course of seed development were present in clusters 78–105, which showed a common pattern of up regulation at the last time points (Supplementary Figure 1, Table 2). Some of the genes that are part of this late phase of seed development are well known as components of stress adaptation in other plant tissues, such as the LEA and heat shock proteins [53,54,55]. Representatives from these gene families (91–94: Glyma13g16510, Glyma01g39260, Glyma07g04860, Glyma16g01440, Glyma03g31380, Glyma08g26100, Glyma19g32920, 100-104: Glyma07g02480, Glyma08g23540, Glyma07g32070, Glyma11g37450, and Glyma16g23750) are present in the late clusters of this data set.

**Table 2:** Soybean genes expressed during the dormancy induction phase in clusters D and E (see Figure 4). Arabidopsis homologs of soybean genes expressed during dormancy induction were categorized by search terms present in GO biological process categories. Each cluster group has the number of Arabidopsis homologs with GO annotations and the percentage of genes matching the search term in the left most column. Gene percentages are calculated using the number of search term matches in the cluster divided by the total genes in the cluster. ‘All clusters’ include both cluster D and E. Each cluster contains unique genes with no overlap, but a gene may be present in more than one category and the total number of genes represents non-redundant genes. All gene percentages > 10% are highlighted in bold.

GO Search Terms	Trend D		Trend E		Both Trends (D + E)	
	Number	%	Number	%	Number	%
abscisic acid	19	7.36	16	8.56	35	8.05
ethylene	9	3.49	11	5.88	20	4.60
jasmonic acid	16	6.20	13	6.95	29	6.67
salicylic acid	14	5.43	13	6.95	27	6.21
chloroplast	8	3.10	1	0.53	9	2.07
redox	4	1.55	3	1.60	7	1.61
germination	7	2.71	1	0.53	8	1.84
flowering	5	1.94	1	0.53	6	1.38
dormancy	9	3.49	6	3.21	15	3.45
transcription	37	**14.34**	28	**14.97**	65	**14.94**
signaling	28	**10.85**	21	**11.23**	49	**11.26**
metal	5	1.94	4	2.14	9	2.07
iron	8	3.10	2	1.07	10	2.30
trehalose	0	0.00	2	1.07	2	0.46
stress	36	**13.95**	24	**12.83**	60	**13.79**
oxidative stress	11	4.26	9	4.81	20	4.60
salt stress	20	7.75	9	4.81	29	6.67
osmotic stress	6	2.33	4	2.14	10	2.30
biotic stimulus	2	0.78	3	1.60	5	1.15
defense response	24	9.30	34	**18.18**	58	**13.33**
water deprivation	17	6.59	13	6.95	30	6.90
Total number of genes	258		187		435	

Transcriptional and post-transcriptional events occur in drying and dry seeds [56] and these represent events that have the potential to affect germination and emergence. Further evidence for this is afforded in our data set, in which soybean genes encoding members of several groups of transcription factors (37 in clusters 91–94 and 28 in clusters 100–104), and genes associated with signal transduction events (28 in clusters 91–94 and 21 in clusters 100–104), are specifically up regulated at the last two time points of seed development. The acquisition of desiccation tolerance involves the production of stored mRNAs, whose encoded proteins are essential for subsequent germination [57]. Evidence for this association between transcriptional events occurring during the final phases of seed maturation and those occurring during the early stages of germination is afforded by the expression of several genes associated with germination in Clusters 91–94 (7 genes: Glyma04g09380, Glyma11g02040, Glyma06g09520, Glyma13g39090, Glyma17g14650, Glyma17g36080, and Glyma04g40880) and 100–104 (Glyma17g09850).

As can be seem from the results presented here, seed maturation and dormancy involve much transcriptional activation and signaling right through the maturation period, especially via the ethylene and ABA pathways. An active role appears to be being played by the chloroplast as a center of metabolic activity. This latter result is somewhat surprising as the chloroplast is losing its photosynthetic capacity during the later stages of seed development, when chlorophyll loss appears to be already taking place.

### 2.3. Integrated Overview of Transcriptional and Metabolic Changes Representing Developmental and Metabolic Transitions during Soybean Embryo Development

A systems biology global perspective for viewing the interacting cellular components (genes, transcripts, proteins, and metabolites) and their regulatory networks offers unprecedented insights into their cellular functions at the molecular level [32,58,59]. Metabolite and transcript profiling are high-throughput systems biology approaches that enable the assessment of steady-state metabolite and transcript levels, respectively, at a particular moment in the studied system [33,60,61]. Current trends in systems biology high-throughput approaches involve tissue- and cell-specific analyses to obtain a global view of processes specific to the studied system [62,63,64]. The major problem in achieving meaningful results from such analyses is the ability to isolate or separate specific cells or tissues of interest without altering the corresponding *in situ* transcriptomes, proteomes, and metabolomes [65].

Developing soybean embryos appear to represent a unique and highly specific system from this perspective, as the majority of the embryo biomass is represented by cotyledons, with only a limited number of cell types and with the majority of embryonic cells in the cotyledon involved primarily in central carbon and nitrogen metabolism specific to seed filling and desiccation [16,19]. However, as the embryos mature and become dense with seed storage compounds, gradients of different types of metabolism occur in layers of cells with different light and oxygen levels as observed for developing soybean embryos and barley endosperm [12,66]. As such, transcript and metabolite profiling performed on whole embryos does not provide information on gradients within the embryo or subcellular localization of metabolites. Although the transcriptional and metabolic changes discussed here correspond to convoluted metabolic and regulatory processes within the whole embryo, they provide valuable information needed for metabolic engineering.

Cells of developing embryos undergo transcriptional and metabolic reprogramming during two main transitions between different types of development and metabolism. First, dividing and differentiating embryonic cells progressively switch their developmental program to cell elongation at the onset of the seed filling phases. This developmental switch is accompanied by gradual metabolic changes from heterotrophic metabolism providing substrates and energy for cell division and differentiation in non-green embryos to photoheterotrophic metabolism during the seed storage reserve accumulation phases. Second, elongating cells at the seed-filling stage turn on seed maturation and desiccation processes to prepare seeds for dormancy and photoheterotrohic metabolism transitions to heterotrophic one. We were able to capture transcriptional and metabolic changes at the end of the first transition in already green embryos, cells of which underwent a combination of cell division and elongation as well as the beginning of the second transition in the elongating cells of yellowing embryos. This enabled the identification of genes potentially connected to developmental, metabolic, and regulatory processes in seed-filling and desiccation phases.

Embryos at early stages of seed filling (days 5 to 15) are already green and accumulating seed storage reserves. From the developmental perspective, these fully differentiated young embryos undergo a combination of cell division and elongation, as numerous mitotic cell-cycle-related structural and regulatory genes, including microtubule-based molecular movement, DNA replication, chromosome remodeling, and epigenetic regulation, were still expressed in the beginning of seed filling. However, their relative steady-state transcript levels decreased rapidly within the first 10 days, suggesting that the sole cell elongation starts between day 10 and 15 in the time course (22- to 32-day-old embryos) during seed filling. From the metabolic perspective, these young embryos also accumulated very high levels of the precursors of seed storage compounds including carbohydrates, carboxylic acids, and amino acids. The levels of these metabolic intermediates became gradually depleted, which also coincided with a similar decrease in the transcript levels of many metabolic genes involved in various aspects of central carbon and nitrogen metabolism, including glycolysis, citric acid cycle, pentose-phosphate pathway, fatty acid synthesis, amino acid metabolism, starch metabolism, cell wall remodeling, and metabolite transport. However, we cannot rule out a possibility that other components of embryo biomass that were not measured would show trends correlating with the trends of these genes and metabolites. Clusters 21–26 (trend B) were particularly enriched in these metabolic genes. Because their expression coincided with the initial decreases in metabolite levels, we hypothesized that these genes encode enzymes involved in metabolism during early embryogenesis. This type of metabolism remains largely unexplored, as early embryo development has been extensively studied from the developmental, rather than a metabolic perspective [67,68,69,70]. As such, the predicted involvement of these genes in central carbon and nitrogen metabolism remains to be confirmed experimentally.

A similar set of metabolic genes showed a nearly opposite trend (clusters 59–62, trend C), as their expression increased during seed filling, while the precursors of seed storage compounds, especially amino acids, became nearly depleted. Gradual initial decreases in the levels of these intermediates of central carbon and nitrogen metabolism correlated well with the increase in the levels of total protein and oil-derived fatty acids. It appears that the levels of metabolic intermediates are kept high in cells undergoing active mitotic cell division. Metabolism occurring during cell division needs to support DNA synthesis, transcription and translation to generate new proteins, membrane lipid synthesis, cell-wall synthesis and remodeling, and synthesis of other cellular components. This represents “an objective” different from metabolism needed for cell elongation and seed filling. Metabolism during seed filling is very demanding for metabolic intermediates, as these are the stages when the embryos gain the most biomass. A possible explanation for the observed gradual decrease in amino acids, monosaccharides, and citric acid cycle intermediates during the mixed cell division and elongation stage is that these metabolites are quickly used for the synthesis of seed storage compounds as the embryos transition to seed filling. Levels of most metabolic intermediates are low during seed filling. Metabolite levels do not reflect metabolic activity of the system and low metabolite levels especially in central carbon and nitrogen metabolism are often indicative of very large metabolic fluxes producing and consuming these metabolites [16,19,71,72,73]. Although these correlations may be coincidental, our gene expression data are consistent with very active central carbon and nitrogen metabolism observed during seed filling in developing soybean embryos [11,16,18,19]. Genes present in clusters 59–62 represent potential targets for metabolic engineering of seed storage compounds.

## 3. Experimental Section

### 3.1. Plant Growth and Embryo Harvesting

Soybean seeds of *Glycine max* (L.) Merr. *cv*. Williams 82 were potted in ProMix Sunshine #1 in 2-gallon pots and grown under controlled growth chamber conditions, as follows: 11/13 hour day/night photoperiod, 28/22 °C day/night temperatures with light intensities between 350 and 450 μE and the relative humidity 70%. Six plants (to provide 3 biological replicates for each time point, with each replicate represented by embryos pooled from two plants) were grown for 30–35 days to achieve the early R5 stage that is characterized by having the first pod containing seeds that are 3 mm long. Seed length was measured with a ruler over the pod on one side while light was shed from the other side to highlight the shape of the seed inside the pod. Every pod that met the 3-mm length criteria was tagged as “day 0” of the time course by color-marking the tip of the pod. The pods were harvested (from branches of similar height to ensure exposure to similar light levels) on ice randomly at 5-day intervals (except for the 10-day interval for the last time point) from the day they were tagged throughout the development from the early R5 to the early R7 stages (transition from green to yellow seeds), yielding the embryos belonging to days 5, 10, 15, 20, 25, 30, 35, 40, 45, and 55. Dissected embryos were rinsed in ice-cold water, immediately frozen in liquid nitrogen and stored at −70 °C until embryos from all time points were harvested before grinding and extractions. Frozen embryos were ground to a fine powder. For biomass measurements and metabolite profiling, the powder was lyophilized for 3 days and the measurements were performed on 1.00 ± 0.05 mg of the dry weight. Frozen powdered tissue from the same experiment was used for RNA extractions.

### 3.2. Biomass Measurements

Lipids and proteins were extracted from the lyophilized powder with heptanes, diethyl ether, and water of equal volumes (400 μl each) in the presence of 10 μg heptadecanoic acid as an internal standard for fatty acid analysis. *(A) Fatty acid analysis.* Fatty acids were analyzed after acidic hydrolysis as fatty acid methyl esters (FAME) by gas chromatography coupled with flame ionization detection (GC-FID) as described [74]. FAME separation and analysis was achieved on an Agilent 7890A series GC-FID (Agilent Technologies, Santa Clara, CA, USA) equipped with a 30-m DB-23 column (0.25 mm × 0.25 μm, Agilent Technologies). *(B)* *Total protein analysis.* Total proteins present in the aqueous phase and insoluble interphase were solubilized in the presence of 0.1% (w/v) sodium dodecylsulfate and their relative levels measured using the MGT hydrophobic protein assay kit (Marker Gene Technologies, Eugene, OR, USA) on a BioTek Synergy™ H4 plate reader (BioTek, Winooski, VT, USA) following the manufacturer recommendations.

### 3.3. Metabolite Profiling

Untargeted polar metabolite profiling was performed as described previously [75,76,77]. Briefly, the lyophilized powder was extracted with 400 μL each chloroform and water containing norvaline and ribitol (50 μM each) as internal standards. Aqueous phase (100 μl) containing polar metabolites was dried under a stream of nitrogen gas at 55 °C and the metabolites were derivatized first with methoxyamine.HCl and then with 2,2,2-trifluoro-n-methyl-n-(trimethylsilyl)-acetamide containing 1% trimethylchlorosilane (Thermo Fisher Scientific, Waltham, MA). Trimethylsilylated metabolite derivatives were separated on an Agilent 7890A series GC equipped with a DB-5MS-DG column (30 m length × 0.25 mm × 0.25 μm with a 10-m pre-column, Agilent Technologies) and analyzed on an Agilent 5975C series single quadrupole mass spectrometer (MS). The GC temperature program and MS conditions were as described [78], except that the m/z scan range was from 100 to 650. Metabolites were identified using the FiehnLib spectral and retention time library [78], our own custom-built spectral and retention time library, and the spectral NIST library (National Institute of Standards and Technology, Gaithersburg, MD). Automated Mass Spectrometry Deconvolution and Identification System (AMDIS, NIST) was used to deconvolute signals from the coeluting compounds. We were able to identify and quantify the relative levels of 55 major metabolites in developing soybean embryos by using the Enhanced Mass Selective Detector ChemStation software (Agilent Technologies) in combination with the three above-mentioned libraries. The identities of metabolites and quality of integration were curated manually on an individual basis by using the QEdit function of the ChemStation software after automated peak area integration. Relative areas of the internal standards were used to correct for recovery in quantitation of each metabolite and all samples were standardized in respect to the dry weight of the tissue used for extractions.

Most amino acids in embryos at or after day 20 could not be detected by the GC-MS-based metabolite profiling. As such, their absolute levels in all collected embryos were determined by using an H-class Acquity UPLC coupled to a fluorescent detector (FLD) (Waters, Milford, MA). Amino acids were extracted with equal volumes (100 μL) of chloroform and 10 mM HCl containing 20 μM norvaline as an internal standard. Five and ten μL of the aqueous phase from first two and the remaining time points, respectively, were taken to determine the levels of free amino acids in a 0.5-μL injection (50-μL total reaction volume) by using the AccQ•Tag Ultra Amino Acid kit (Waters) according to the manufacturer’s recommendations. Two different UPLC gradients were used to enable the separation of (i) Gln and Asn and (ii) Glu and citrulline. The first gradient method used was developed by Waters for analysis of free amino acids in cell cultures and was implemented without any modifications. The second method was based on another Waters method originally intended for analyzing amino acids originating from protein hydrolysates, but with modifications to the UPLC gradient to at least partially separate Glu and citrulline. The following gradient with Waters Eluent A (5% in water by volume) and undiluted Eluent B at a flow rate of 0.7 ml min^−1^ were used (% given for A and curve was 6 unless otherwise stated): 0–0.54 min isocratic 99.9%; 5.74 min 90.9% (curve 7); 7.74 min 78.8%; 8.04 min 40.4%; 8.05–8.64 min isocratic 10%; 8.65–9.00 min isocratic 0%; and 9.20–12.00 min equilibration to 99.9%. Standard curves and Empower 3 software (Waters) were used to obtain absolute amino acid levels (corrected for recoveries and dry weight) in developing soybean embryos.

### 3.4. Transcriptomics

#### 3.4.1. RNA Isolation, cDNA Library Preparation, and Illumina RNA Sequencing

RNA sequencing was performed to investigate changes in the soybean transcriptome during seed development. First, total RNA was isolated from the frozen ground embryos by using an RNeasy Plant RNA Purification Mini Kit (Qiagen, Germantown, MD) according to the manufacturer’s recommendations, with a minor modification. The samples were centrifuged at 16,000 g at 4 °C for 2 min after the addition of the RLT buffer to obtain a clear aqueous phase for the subsequent column purification steps. The RNA concentration and quality was measured by using a Bioanalyzer 2100 (Agilent Technologies). A minimum of 400 ng mL^−1^ of RNA and an RNA integrity number (RIN) greater than 8.0 was obtained for reliable RNA sequencing analyses. Library preparation and RNA sequencing analysis were performed at the Génome Québec Innovation Centre (Montréal, Canada). The cDNA libraries were generated using TruSeq RNA sample preparation kit (Illumina, San Diego, CA) and paired-end 100-bp long RNA-seq reads were generated on a HiSeq 2000 sequencing system (Illumina), with each lane containing multiplexed cDNA libraries pooled from the three biological replicates of the individual time-points.

#### 3.4.2. RNA Sequencing Data Processing, Differential Gene Expression, and Gene Coexpression Pipeline

**Figure 5:**
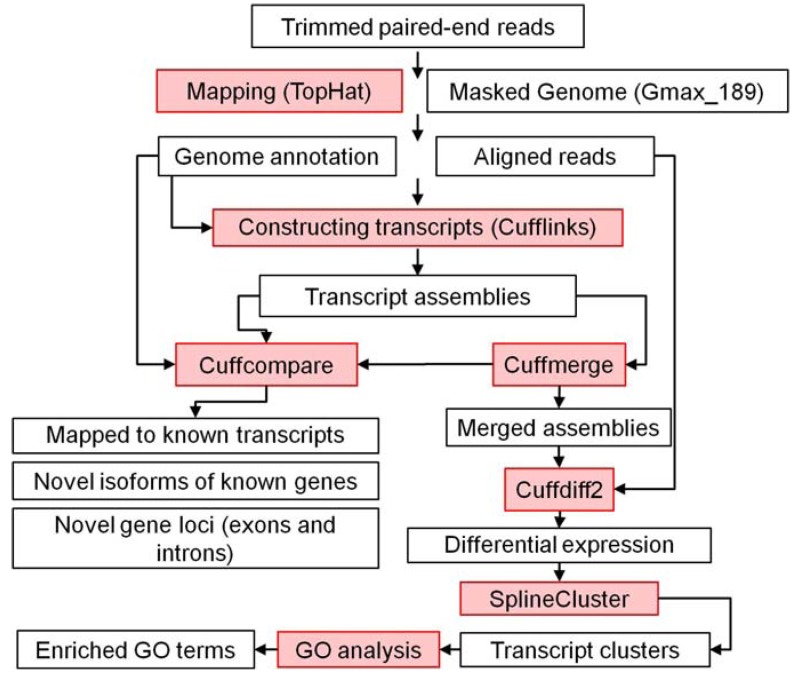
RNA sequencing, differential expression, and coexpression pipeline. Computational tools are shown in color.

Our pipeline (Figure 5) consists of the following steps: First, each sequence read was evaluated for the presence of a recognizable, library-specific 5’ barcode sequence. Raw reads with length ≤32 and the quality score ≤30 were filtered out using Illumina purity filter and further evaluated based on the distribution of phred-like quality scores at each cycle. The demultiplexed reads were then mapped to the reference genome using Tophat, which internally uses Bowtie as its high-throughput read alignment tool. Tophat can also find splice junctions between exons with good accuracy [79]. The resulting reads were mapped to version 1.0 of the *G. max* reference genome [40] provided in Phytozome v8.0 [31] using TopHat v2.0.4 [80] in conjunction with Bowtie v0.12.8 [81] using all default parameters except for the distance between mated pairs set to 60 bp. Results providing statistical information about raw, filtered, and aligned reads are provided in Table 3.

Second, the reads were concatenated using Cufflinks [80]. The RABT (Reference Annotation Based Transcript) assembly technique was used for this purpose [82], which results in a good accuracy for finding novel genes and isoforms of genes when a high quality sequence reference exists for that genome. Three transcript assemblies in each sample from Cufflinks results were merged using the Cuffmerge tool [83]. The Cufflinks and Cuffmerge results were compared with the reference genome using Cuffcompare to find known genes, novel genes, novel splice variants, and transcripts expressed from intergenic regions [83]. Information about the read alignment and transcript assembly statistics is presented in Supplementary Tables 6 and 7.

Third, the reads from Tophat and merged assemblies from Cuffmerge were fed to Cuffdiff2 [83] for testing differential expression in a time-series manner, including splice variants. This allows testing for the presence of differentially-expressed genes between two consecutive time points and the identification of gene sets showing statistically significant change (p-value < 0.05) at least in one of the time points (10794 genes). These differentially-expressed genes were subjected to the SplineCluster clustering software enabling gene coexpression analysis in a time-series-dependent manner [28]. Since we were interested in common patterns of behavior for genes across the time course (not the actual expression values), the mean of each gene expression value was moved to a fixed value before the clustering analysis. The lowest gene expression value was 4.71126E-11 and all zeroes were replaced with this value to prevent the infinity problem (dividing by zero) to enable differential gene expression analysis. They were considered not to be expressed. The Arabidopsis homologs of the clustered soybean genes were associated with GO annotations with an in-house perl program using the Gene Ontology OBO v1.2 specification and The Arabidopsis Information Resource (TAIR) GO annotations (both downloaded from their respective websites on March 11, 2013). GEO accession number for this RNA sequencing data is GSE46153.

**Table 3:** Statistical results from RNA sequencing analysis (number and percentage of raw, filtered, and aligned reads). Three replicates (a, b, and c) are shown for each time point (days 5–55).

sample	raw	%	filtered	%	aligned	%
d5a	50,277,367	100	48,847,168	97.2	45,036,342	89.6
d5b	62,438,141	100	60,618,219	97.1	55,916,016	89.6
d5c	42,789,850	100	41,586,412	97.2	38,479,330	89.9
d10a	72,420,225	100	69,364,427	95.8	61,431,613	84.8
d10b	46,641,758	100	44,850,498	96.2	39,812,742	85.4
d10c	85,142,664	100	81,774,580	96.0	71,198,971	83.6
d15a	42,701,829	100	39,342,420	92.1	33,742,148	79.0
d15b	55,919,488	100	51,665,677	92.4	44,507,297	79.6
d15c	98,613,720	100	91,253,129	92.5	78,873,912	80.0
d20a	71,059,794	100	65,828,827	92.6	52,933,436	74.5
d20b	44,455,535	100	41,248,905	92.8	33,195,196	74.7
d20c	54,423,534	100	50,618,077	93.0	40,743,929	74.9
d25a	61,500,744	100	56,010,032	91.1	46,786,403	76.1
d25c	83,670,143	100	76,387,754	91.3	63,785,353	76.2
d30a	74,112,923	100	68,885,120	92.9	59,572,944	80.4
d30b	77,985,515	100	72,428,008	92.9	61,711,555	79.1
d30c	100,671,683	100	93,523,791	92.9	81,026,533	80.5
d35a	67,498,080	100	62,861,610	93.1	54,691,875	81.0
d35b	87,943,727	100	82,007,125	93.2	71,352,895	81.1
d35c	99,568,258	100	92,761,463	93.2	80,856,912	81.2
d40a	135,660,811	100	124,626,322	91.9	107,084,232	78.9
d40b	54,135,432	100	49,929,189	92.2	43,065,040	79.6
d40c	55,241,634	100	51,064,159	92.4	44,214,195	80.0
d45a	67,554,585	100	63,069,990	93.4	53,773,223	79.6
d45b	62,187,743	100	58,293,335	93.7	50,756,256	81.6
d45c	74,873,986	100	70,329,261	93.9	60,877,029	81.3
d55a	74,383,353	100	68,110,555	91.6	56,475,935	75.9
d55b	86,213,964	100	79,106,644	91.8	65,108,144	75.5
d55c	47,160,359	100	43,338,248	91.9	35,914,805	76.2

#### 3.4.3. MapMan

Modified MapMan that enables the visualization of time-courses within specific bins [29,30,42] was used to visualize possible temporal changes in gene expression. To generate the MapMan input file, FPKM gene expression values obtained from Cuffmerge output for every gene at every time point were compared to the corresponding values in the first time point to obtain fold-change in gene expression. We used log_2_(fold-change) relative to the first time point and only genes present in the MapMan Gmax_109_peptide mapping file and showing a statistically significant change (*p* ≤ 0.05) for at least one time point were included in the MapMan input file for visualization purposes. Soybean gene IDs did not contain alternate splicing information and as such, the MapMan Gmax_109_peptide mapping file was altered to remove this information as well as all redundant gene ID entries and the resulting Gmax_109_peptide_mod mapping file is available upon request.

## 4. Conclusions

We generated high quality transcriptomic and metabolomic data relevant to seed filling and desiccation acquisition in developing soybean embryos. Computational analyses of these large datasets enabled a systems biology view of global transcriptional and metabolic changes during transitions from cell division to elongation and from seed filling to desiccation processes. From the metabolic engineering perspective, there appears to be a set of metabolism-related genes specific to metabolism at early stages of soybean embryo development and another set of similar genes expressed during seed filling. These genes represent potential targets for future metabolic engineering of seed composition. However, there is a high level of genetic and metabolic redundancy in developing soybean embryos. In addition, central carbon and nitrogen metabolism is highly regulated and there are several levels of regulation between transcription and the final product/process targeted for metabolic engineering.

Detailed coexpression analysis in combination with metabolomic data presented in this study also provide a valuable resource of potential regulators associated with metabolic and desiccation tolerance genes in individual clusters. Every cluster contained a number of transcription factors, protein kinases and phosphatases, components of the ubiquitin-associated protein degradation system, various proteases, and genes encoding other protein-modifying enzymes such as farnesyl and acyl transferases. Besides regulators, our datasets enabled the identification of splice variants and at least 3400 novel genes containing exons and introns that map to the soybean genome for future studies. 

The relevant responses underlying desiccation acquisition or drought tolerance have historically been regarded as stress related. In the case of developing seeds, this genetically programmed developmental process proves to be more complex than a simple acquisition of desiccation tolerance and accumulation of raffinose-related oligosaccharides. As can be seen from the results presented here, seed maturation and dormancy involve much transcriptional activation and signaling right through the maturation period, especially via the ethylene pathway with an active role being played by the chloroplast as a center of metabolic activity. This latter result is somewhat surprising as the chloroplast is losing its photosynthetic capacity during the later stages of seed development. As such, understanding the underlying mechanisms for these responses is potentially useful in engineering drought and desiccation tolerant plants.

## Supplementary Files

Supplementary File 1 Supplementary (ZIP, 15341 KB)
