# Supplementary material for: Metabolic and Transcriptional Reprogramming in Developing Soybean (Glycine max) Embryos
Source: Metabolites. 2013 May 14;3(2):347–72. doi: 10.3390/metabo3020347 (PMC3901275; doi:10.3390/metabo3020347)
Supplement: Supplementary File 1 — Supplementary (ZIP, 15341 KB) [file metabolites-03-00347-s001.zip › metabolites-03-00347-supplementary-final format/Supplementary Content.docx]

**Supplementary Content**

**Video. Log_2_ fold changes in metabolite and transcript levels during embryo development visualized using “Metabolism Overview” in MapMan.** The heatmap scale was set to ± 10. Blue represents decreased and red increased transcript and metabolite levels compared to the first time point (day 5). Only statistically significant changes are displayed (MapMan cutoff for the *p*-value was set to 0.05).

**Supplementary Figure 1. Expression profiles of responsive genes (10794) present in the 105 clusters generated by SplineCluster.** The individual gene expression profiles are shown as black lines with the mean expression values in dark blue. The y-axes indicate the normalized log_2_(fold-change gene expression values) and the x-axes indicate time (days after pod tagging). The scale in all clusters is the same.

**Supplementary Table 1. Metabolite levels in developing soybean embryos.** Relative metabolite levels were determined by GC-MS, while the absolute levels of amino acids and organic amines were determined by UPLC-FLD as described in the Experimental Section. Measurements of 3 averaged replicates (AVG) and standard deviation (SD) are shown next to each other for days 5 – 55 for each metabolite. Both retention time and spectral information were used on deconvoluted spectra of metabolites for metabolite identification and “?” placed after the names of some metabolites indicates that the identity of the metabolite was ambiguous.

**Supplementary Table 2. Differential expression of genes during soybean seed development.** All 10794 genes that showed significant differential expression (p-value < 0.05) in at least one of the time points compared with the previous one (obtained from Cuffdiff2) are presented as well as the corresponding expression values (FPKM) and *p*-values for each comparison

**Supplementary Table 3. Soybean MapMan input file.** This file was used as an input file containing gene expression data for developing soybean embryos in MapMan Metabolism Overview video. Each gene (represented by Glyma gene I.D.) is in column A. The following two consecutive numbers correspond to the log_2_(fold change) gene expression value compared to the first time point (day 5) and the corresponding *p*-values, respectively. For example, “d15_d5_fold_change” means that this value corresponds to log_2_(FPKM value for day 15/FPKM value for day 5). The number next to it under “d_d15_d5_fold_change” is the corresponding *p*-value.

**Supplementary Table 4. SplineCluster coclustering analysis of genes that showed changes in gene expression during soybean seed development.** All 105 clusters, their corresponding genes, the closest Arabidopsis homologues and MapMan annotations are shown.

**Supplementary Table 5. Clusters containing genes related to seed dormancy and acquisition of desiccation tolerance in developing soybean embryos.** Clusters 91 – 94 and 100 – 104 are shown along with the MapMan bincode, soybean gene ID, MapMan annotation, gene I.D. of the closest Arabidopsis homolog, GO biological process annotations, GO cellular component annotations, and GO molecular function annotations.

**Supplementary Table 6. Mapping to the reference genome and read statistics.** Mapping statistics to the reference genome (total number of reads (read1 + read 2), total number of paired reads, singletons, and spliced reads and their percentages) was performed using SAM tools [1]. All reads were mapped successfully to the reference genome and were 100% paired in sequencing as well. Among all mapped reads, the majority (77% on average) were “properly paired”, which means that both mates of a read pair mapped to the same chromosome, and were oriented towards each other (“with itself and mate mapped” in the Table) with insert size = 3 (default). Approximately 7% of reads were scored as singletons. Approximately 26% of all reads were mapped to known or novel splice junctions.

**Supplementary Table 7. Transcript assembly statistics.** Transcript assembly statistics were obtained by using the Cuffcompare tool. Number of matching intron chains (MIC), matching loci (ML), total loci, number and percentage of missed exons, novel exons, missed introns, novel introns, missed loci, and novel loci are shown. Cuffcompare compares assembled transcripts to the reference annotation and reports different statistics related to the accuracy of the assembled transcripts. The soybean reference annotation had 55756 mRNAs in 46386 loci. On average, 451144 loci matched to the loci in the reference genome, and about 47645 introns matched to the intron chains. Missed exons are defined as the proportion of true exons with no overlap to predicted exons (0.15% on average). Novel exons are the proportion of predicted exons without overlap to actual exons (7.27% on average). Similar definitions apply for introns and loci.

1. Li, H.; Handsaker, B.; Wysoker, A.; Fennell, T.; Ruan, J.; Homer, N.; Marth, G.; Abecasis, G.; Durbin, R. The Sequence Alignment/Map format and SAMtools*.* *Bioinformatics* **2009**, *25*, 2078-2079.
