# Supplementary material for: Metabolic and Transcriptional Reprogramming in Developing Soybean (Glycine max) Embryos
Source: Metabolites. 2013 May 14;3(2):347–72. doi: 10.3390/metabo3020347 (PMC3901275; doi:10.3390/metabo3020347)
Supplement: Supplementary File 1 — Supplementary (ZIP, 15341 KB) [file metabolites-03-00347-s001.zip › metabolites-03-00347-supplementary-final format/Supplementary Table6.docx]

**Supplementary Table 6. Mapping to the reference genome and read statistics.** Mapping statistics to the reference genome (total number of reads (read1 + read 2), total number of paired reads, singletons, and spliced reads and their percentages) was performed using SAM tools [1]. All reads were mapped successfully to the reference genome and were 100% paired in sequencing as well. Among all mapped reads, the majority (77% on average) were “properly paired”, which means that both mates of a read pair mapped to the same chromosome, and were oriented towards each other (“with itself and mate mapped” in the Table) with insert size = 3 (default). Approximately 7% of reads were scored as singletons. Approximately 26% of all reads were mapped to known or novel splice junctions.

1. Li, H.; Handsaker, B.; Wysoker, A.; Fennell, T.; Ruan, J.; Homer, N.; Marth, G.; Abecasis, G.; Durbin, R. The Sequence Alignment/Map format and SAMtools*.* *Bioinformatics* **2009**, *25*, 2078-2079.
