# Supplementary material for: Metabolic and Transcriptional Reprogramming in Developing Soybean (Glycine max) Embryos
Source: Metabolites. 2013 May 14;3(2):347–72. doi: 10.3390/metabo3020347 (PMC3901275; doi:10.3390/metabo3020347)
Supplement: Supplementary File 1 — Supplementary (ZIP, 15341 KB) [file metabolites-03-00347-s001.zip › metabolites-03-00347-supplementary-final format/Supplementary Table7.docx]

Supplementary Table 7. Transcript assembly statistics. Transcript assembly statistics were obtained by using the Cuffcompare tool. Number of matching intron chains (MIC), matching loci (ML), total loci, number and percentage of missed exons, novel exons, missed introns, novel introns, missed loci, and novel loci are shown. Cuffcompare compares assembled transcripts to the reference annotation and reports different statistics related to the accuracy of the assembled transcripts. The soybean reference annotation had 55756 mRNAs in 46386 loci. On average, 451144 loci matched to the loci in the reference genome, and about 47645 introns matched to the intron chains. Missed exons are defined as the proportion of true exons with no overlap to predicted exons (0.15% on average). Novel exons are the proportion of predicted exons without overlap to actual exons (7.27% on average). Similar definitions apply for introns and loci.
