# Supplementary figures and images for: Metabolic and Transcriptional Reprogramming in Developing Soybean (Glycine max) Embryos
Source: Metabolites. 2013 May 14;3(2):347–72. doi: 10.3390/metabo3020347 (PMC3901275; doi:10.3390/metabo3020347)

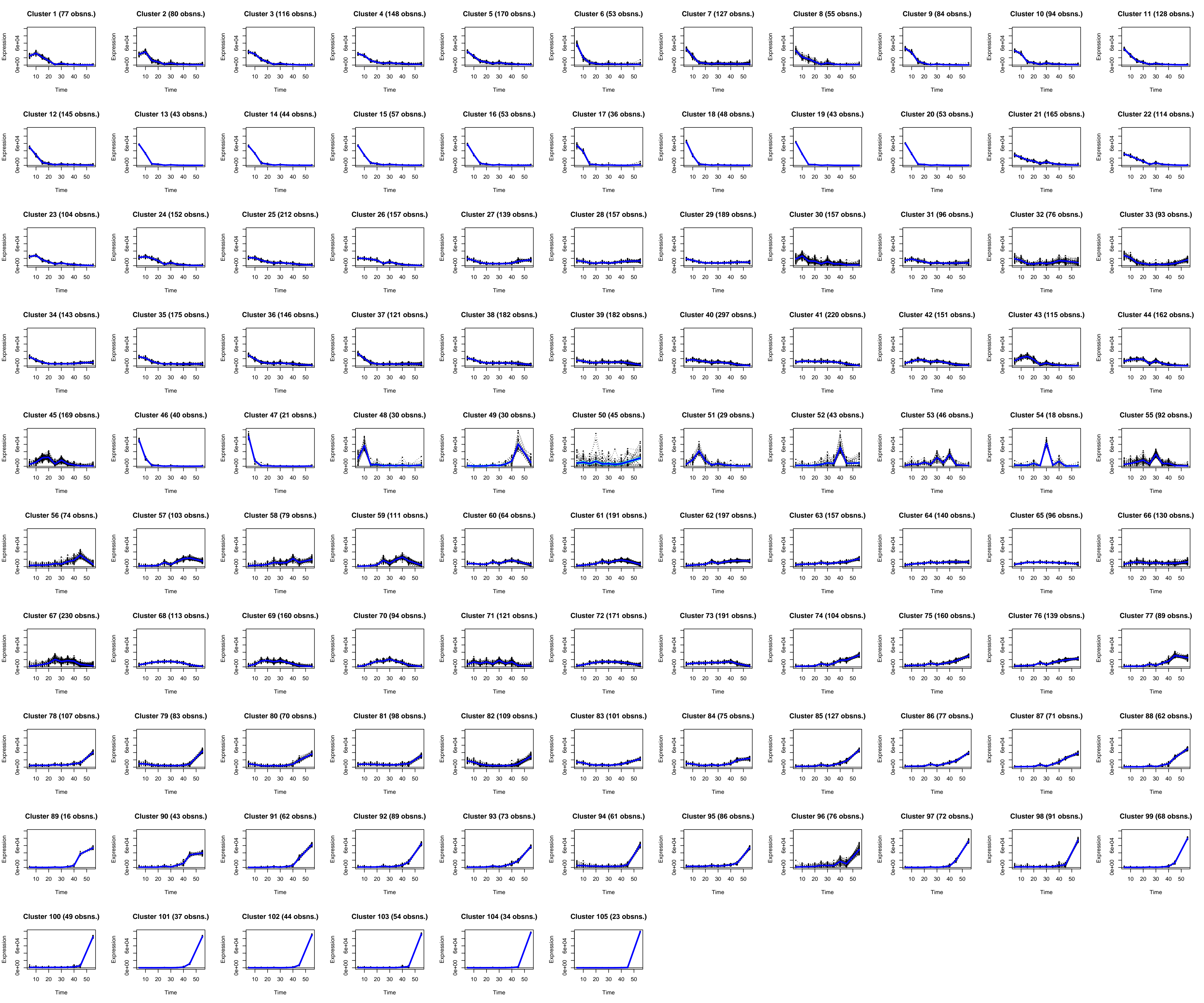

Supplement: Supplementary File 1 — Supplementary (ZIP, 15341 KB) [file metabolites-03-00347-s001.zip › metabolites-03-00347-supplementary-final format/Supplementary Figure1.pdf]
